# Role of LARP6 and Nonmuscle Myosin in Partitioning of Collagen mRNAs to the ER Membrane

**DOI:** 10.1371/journal.pone.0108870

**Published:** 2014-10-01

**Authors:** Hao Wang, Branko Stefanovic

**Affiliations:** Department of Biomedical Sciences, College of Medicine, Florida State University, Tallahassee, Florida, United States of America; University of Toronto, Canada

## Abstract

Type I collagen is extracellular matrix protein composed of two α1(I) and one α2(I) polypeptides that fold into triple helix. Collagen polypeptides are translated in coordination to synchronize the rate of triple helix folding to the rate of posttranslational modifications of individual polypeptides. This is especially important in conditions of high collagen production, like fibrosis. It has been assumed that collagen mRNAs are targeted to the membrane of the endoplasmic reticulum (ER) after translation of the signal peptide and by signal peptide recognition particle (SRP). Here we show that collagen mRNAs associate with the ER membrane even when translation is inhibited. Knock down of LARP6, an RNA binding protein which binds 5′ stem-loop of collagen mRNAs, releases a small amount of collagen mRNAs from the membrane. Depolimerization of nonmuscle myosin filaments has a similar, but stronger effect. In the absence of LARP6 or nonmuscle myosin filaments collagen polypeptides become hypermodified, are poorly secreted and accumulate in the cytosol. This indicates lack of coordination of their synthesis and retro-translocation due to hypermodifications and misfolding. Depolimerization of nonmuscle myosin does not alter the secretory pathway through ER and Golgi, suggesting that the role of nonmuscle myosin is primarily to partition collagen mRNAs to the ER membrane. We postulate that collagen mRNAs directly partition to the ER membrane prior to synthesis of the signal peptide and that LARP6 and nonmuscle myosin filaments mediate this process. This allows coordinated initiation of translation on the membrane bound collagen α1(I) and α2(I) mRNAs, a necessary step for proper synthesis of type I collagen.

## Introduction

Type I collagen is the most abundant protein in human body, found predominantly in skin, bone, tendons and other connective tissues. The protein is composed of two α1(I) polypeptides and one α2 polypeptide. The polypeptides are co-translationally inserted into the lumen of the endoplasmic reticulum (ER), post-translationally modified by hydroxylations and glycosylations, folded into a triple helix and secreted into the extracellular environment [1], [2]. After proteolytic processing, triple helices are polymerized and crosslinked into fibrils [3]. Evidence has been presented that processing and polymerization of collagen helices can take place even in the terminal secretory vesicles [4].

The abundance of type I collagen is primarily due to slow turnover of the protein, rather than due to high rate of synthesis. The half life of type I collagen is about 30–60 days, while its fractional synthesis rate (FSR), expressed as percentage per hour, is estimated to be 0.076±0.063% per hour or about 2% per day [5]. This is substantially slower than, for example, the FSR of plasma proteins, which is about 0.5% per h [6], [7], [8]. However, in reparative [9] or reactive fibrosis [10], [11], [12], [13] the rate of collagen synthesis increases several hundred fold. To enable such outburst of collagen production the cells employ a unique mechanism that is based on binding of LARP6 to the conserved stem loop (SL) found in the 5′ UTRs of mRNAs encoding type I collagen [14], [15]. The collagen 5′SL binds LARP6 with high affinity and specificity and LARP6 recruits accessory factors to promote translation of collagen mRNAs. These factors include RNA helicase A (RHA), FKBP3 and STRAP [16], [17], [18]. RHA promotes loading of polysomes on collagen mRNA, most likely by unwinding the 5′SL [18]. FKBP3 is a chaperone involved in recycling of LARP6 [17], while STRAP is needed for coordination of translation of collagen α1(I) and α2(I) mRNAs [16]. Cells lacking STRAP produce predominantly homotrimers of type I collagen and hypermodified individual α1(I) and α2(I) polypeptides. In addition, LARP6 associates collagen mRNAs with vimentin intermediate filaments to prolong their half life [19]. These processes drive high collagen production in fibrosis.

Nonmuscle myosin is the motor protein which slides actin filaments [20]. The activity of nonmuscle myosin is needed for cell motility, kariokinesis and trafficking of intracellular vesicles and these functions of nonmuscle myosin have been extensively studied [21], [22], [23], [24], [25], [26], [27], [28]. There are three isoforms of nonmuscle myosin, but isoforms IIA and IIB are the most abundant in cells. In fibrosis, quiescent fibroblasts and other cells capable of making type I collagen are activated and differentiate into myofibroblasts [29]. They highly upregulate nonmuscle myosin and acquire the ability to migrate [30], [31], [32]. At the same time they begin to produce large amount of type I collagen. It has been postulated that nonmuscle myosin filaments help secretion of vesicle containing already formed collagen fibers [4]. However, we were first to show that nonmuscle myosin filaments are also needed for synthesis and proper formation of the type I collagen [15], [33].

In this manuscript we extend these observations and show that collagen mRNAs associate with the ER membrane in translation independent manner and that LARP6 and nonmuscle myosin filaments play a role in this process. Direct binding of collagen mRNAs to the ER membrane coordinates translation of collagen mRNAs, resulting in proper modifications of the polypeptides and effective secretion of heterotrimeric type I collagen.

## Material and Methods

### Chemicals

ML-7 (I2764), blebbistatin (B0560), puromycin (P7255), and digitonin (D41) were purchased from Sigma. Pateamine A was purchased from Southwestern Medical Center, Dallas, TX and iodixanol (Opti-prep) was purchased from Accurate Chemical & Scientific CORP (LYS3782).

### Constructs

Dominant negative myosin light chain kinase (DN-MLCK) was a kind gift of Dr. P. Gallagher, Indiana University School of Medicine. The construct was described in [34]. The cDNA of DN-MLCK was recloned into pAdCMV-Track vector and recombinant adenovirus was constructed by recombination in E. coli, as described in [35]. For overexpression of DN-MLCK adenovirus was added at multiplicity of infection of 500 to human lung fibroblasts in culture.

### Cells and transfections

Human lung fibroblasts (HLFs) immortalized by expression of telomerase reverse transcriptase have been described previously [14], [36]. Scleroderma fibroblasts derived from the skin of a scleroderma patients was purchased from European collection of cell cultures (cell line BM0070). Human lung fibroblasts and scleroderma fibroblasts were grown under standard conditions in Dulbecco's modified Eagle's medium supplemented with 10% fetal bovine serum (Valley Biomedical) for up to 10 passages. HEK293T cells were grown in Dulbecco's modified Eagle's medium with 10% of FetalPlus serum for up to 10 passages. For analysis of proteins, ML-7 (40 µM) or Blebbistatin (100 µM) were added 16 hours before the analysis and for analysis of mRNAs they were added 3 hours before the experiment. Pateamine A (200 nM), puromycin (100 µg/ml) or cycloheximide (100 µg/ml) were added 3 hours before the analysis. HEK293T cells were transfected with 1 µg of plasmid per 35 mm dish by 293TransIT reagent (Mirus). LARP6 specific siRNA with the sequence 5′-AGGACGUGCACGAGUUGGAUU-3′ was purchased from Thermo Scientific (Stebo-000001). The non-targeting control siRNA was from Thermo Scientific (D-001210-01-20). HLFs were transfected with siRNA at the final concentration of 150 nM using Lipofectamine 2000 reagent (Invitrogen). The cells were harvested for analysis 48–72 hours after transfections.

### RT-PCR analysis

Total RNA was isolated by Phenol-Chloroform sequential extraction and contaminated DNA was removed by treated with DNase I. 100 ng of total RNA or equivalent amounts (10%) of RNA extracted from cytosolic and membrane fractions were used in RT-PCR. Semi-quantitative RT-PCR reactions were performed using rTth reverse transcriptase (Boca Scientific, FL) and including radio-labeling of the PCR products with 32P-dATP [10], [14], [16], [37], [38]. The primers used are listed in Table 1. To maintain the reaction in the linear range, 23 cycles were used for collagen and fibronectin mRNA. The PCR products were resolved on sequencing gels and specific bands were detected by autoradiography.

**Table 1:** Primers used for RT-PCR and sequences of siRNA.

GENE	FORWARD	REVERSE
COL1A1	AGAGGCGAAGGCAACAGTCG	GCAGGGCCAATGTCTAGTCC
COL1A2	CTTCGTGCCTAGCAACATGC	TCAACACCATCTCTGCCTCG
FIB	ACCAACCTACGGATGACTCG	GCTCATCATCTGGCCATTTT
ACT	GTGCGTGACATTAAGGAGAAG	GAAGGTAGTTTCGTGGATGCC
MMP12	ACACATTTCGCCTCTCTGCT	CCTTCAGCCAGAAGAACCTG
LARP6 siRNA	AGGACGUGCACGAGUUGGAUU	
CON siRNA	D-001210-01-05 (Dharmacon)	

### Real time RT-PCR

RNA from soluble and membrane fractions was isolated by phenol/chloroform extraction and isopropanol precipitation and treated with DNase I to remove contaminating DNA. The first-strand cDNAs were synthesized using SuperScript II RT reverse transcriptase (Invitrogen) and oligo-dT primer using equal amounts (10%) of the soluble and membrane RNA. Quantitative real-time PCR analyses were performed on an IQ5 thermocycler (Bio-Rad) using SYBR green detection kit (Qiagen) and gene specific primers (Table 1). The signals for COL1A1, COL1A2 and MMP12 mRNAs were normalized to signal for actin mRNA. Actin mRNA was found to be equally distributed in soluble and membrane fractions and, therefore, was suitable for normalization. The normalized cytosolic and membrane mRNA signals were added and arbitrarily set as 1 for each gene and the fraction of mRNA in cytosol and membrane was calculated. The experiments were done in three replicates and data are presented as means and standard errors of the mean (SEM).

### Western blots

Total cellular proteins were prepared by lysing cells in 150 mM NaCl, 10 mM MgCl2, 10 mM Tris 7.5 and 0.5% NP-40. The nuclei were removed by centrifugation and protein concentration measured by Bradford assay. Typically, 50 µg of total proteins was used for analysis. For analysis of proteins secreted into cellular medium, equal amount of cells were seeded in 35 mm dishes. After reaching 80% confluency the cells were washed with serum free medium, 500 µl of serum free medium was added and incubation continued for 3 hours. The medium was collected and equal volumes were directly analyzed by Western blotting. The antibodies used were: anti-collagen α1(I) antibody (Rockland, PA); anti- collagen α2(I) antibody (Santa Cruz Biotech, Dallas, Texas); anti-fibronectin antibody (BD Transduction Laboratories, New Jersey); anti-LARP6 antibody (Abnova, CA); anti-tubulin antibody (Cell Signalling, Danvers, MA); anti-calnexin antibody (BD transduction, New Jersey); anti-actin antibody (Abcam, Cambridge, MA); anti-Golgin84 antibody (BD transduction, New Jersey), anti-myosin IIB antibody (Hybridoma bank, University of Iowa) and anti-GAPDH antibody (Santa Cruz Biotechnology). Anti-histone H4 antibody has been described in [39].

### Fractionation of cells into cytosolic and membrane compartments

HLFs were grown to 90% confluency, washed with PBS, gently coated with permeabilization buffer (110 mM KOAc, 25 mM HEPES 7.2, 2.5 mM Mg(OAc)_2_, 1 mM EGTA, 0.015% digitonin, 1 mM DTT, 1 mM PMSF) and rocked slowly on ice for 5 minutes. The soluble cytosolic material was collected and the cell remnants were washed with 110 mM KOAc, 25 mM HEPES 7.2, 2.5 mM Mg(OAc)_2_, 1 mM EGTA, 0.004% digitonin, 1 mM DTT, 1 mM PMSF. The cell membranes were dissolved in 400 mM KOAc, 25 mM HEPES (pH 7.2), 15 mM Mg(OAc)_2_, 1% NP-40, 0.5% DOC, 1 mM DTT, 1 mM PMSF by rocking on ice for 5 minutes and the extracted material was collected [40]. The samples were clarified by centrifugation at 7500 g for 10 minutes at 4°C and used for analysis by Western blot. RNA from the fractions was isolated by phenol-chloroform extraction and isopropanol precipitation. For separation of polysomes the soluble and membrane fractions were loaded onto 25% sucrose cushion and centrifuged at 34,200 rpm for 2 hours at 4°C [40]. The supernatant was collected as post-polysomal supernatant (PPS) and the pellets were dissolved in PBS as polysomes.

### Separation of ER and Golgi compartments

HLFs grown to 80% confluency were treated with ML-7 or DMSO, washed in PBS and homogenized in buffer containing 0.25 M sucrose, 1 mM EDTA, 10 mM HEPES 7.4 using Dounce homogenizer. Nuclei were removed by centrifugation at 3000 g for 10 min and the supernatant was overlaid onto 5% to 25% iodixanol gradient and centrifuged at 34,200 rpm for 2 hours [41]. Sixteen 500 µl fractions were collected from the bottom of the gradient and 80 µl of each fraction was analyzed by Western blot.

### Two-Dimensional Gel Electrophoresis

Proteins were precipitated in 10 volumes of 90% ethanol and protein pellets were solubilized and separated by iso-electric focusing on immobilized 7 cm long pH gradient strips with pH range of 3–10 (GE Healthcare) with total of 5,000 Vh using Ettan IPGphor 3 instrument. The strips were loaded of 7.5% SDS PAGE gels and after separation the gels were blotted and probed with anti-collagen antibody.

## Results

### Translation independent localization of collagen mRNAs to the ER membrane

**Figure 1:**
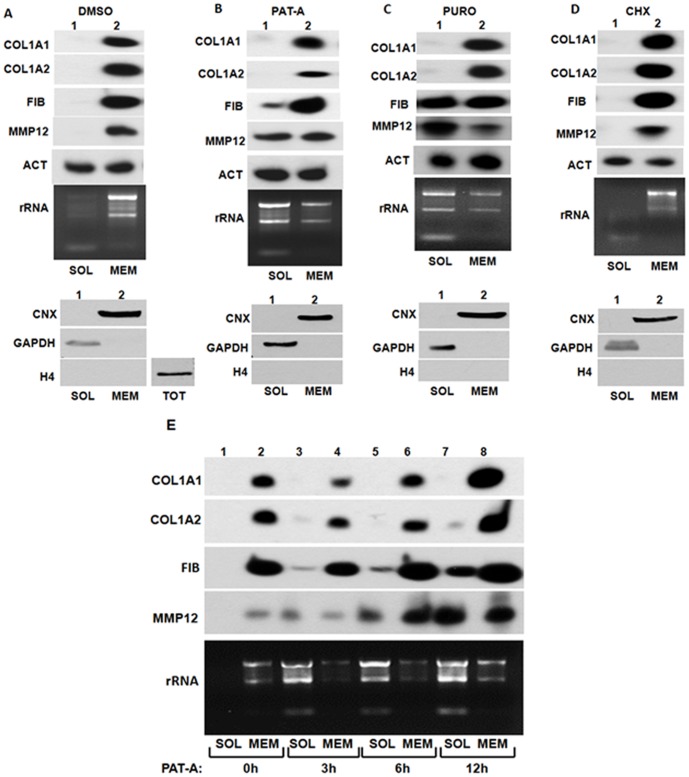
Translation is not required for membrane localization of collagen mRNAs. A. membrane partitioning of collagen mRNAs in control cells. Upper panel: HLFs were fractionated in cytosolic fraction (SOL, lane 1) or membrane fraction (MEM, lane 2) and collagen α1(I) (COL1A1), collagen α2(I) (COL1A2), fibronectin (FIB), matrix metalloproteinase 12 (MMP12) and actin (ACT) mRNAs were analyzed by RT-PCR. Distribution of ribosomal RNA (rRNA) is shown by agarose gel electrophoresis and ethidium bromide staining. Bottom panel: distribution of calnexin (CNX), GAPDH and histone H4 proteins in the fractions, analyzed by western blot. TOT; total cellular extract showing reactivity of the histone H4 antibody. B. Partitioning of collagen mRNAs after translation inhibition by pateamine A (PAT-A). Experiment as in A, except the cells were treated with PAT-A for 3 h prior to harvesting. C. Partitioning after translation inhibition by puromycin. D. Partitioning after translation inhibition by cycloheximide. E. Time course of mRNA redistribution after inhibition of translation. HLFs were treated with PAT-A for the indicated time periods and cells were fractionated into cytosolic (SOL) and membrane (MEM) fractions and the distribution of the mRNAs was analyzed by RT-PCR.

Secreted proteins are translated at the membrane of the ER and co-translationally inserted into the lumen through SEC61 translocation channels [42]. It has been well accepted that the mRNAs encoding secreted proteins are targeting to the ER membrane by signal recognition particle (SRP) after translation of the signal peptide [43]. However, recently it has been shown that many mRNA can associate with the ER membrane in the translation independent manner and it has been postulated that RNA binding proteins mediate this process [44], [45], [46], [47], [48], [49]. Since mRNAs encoding type I collagen bind LARP6 with high specificity [14], we investigated if collagen mRNAs can be targeted to the ER membrane in the absence of translation. To this goal we inhibited translation in human lung fibroblasts (HLF) using three drugs: pateamine A (inhibits translation initiation) [50], [51], puromycin (dissociates polysomes) or cycloheximide (immobilizes polysomes). 3 h after inhibition of translation, we used selective detergent extraction to separate cytosolic RNA and membrane bound RNA [40] and analyzed collagen mRNAs distribution in these fractions. In untreated cells collagen α1(I) and α2(I) mRNAs were found entirely associated with the membrane fraction. The same was true for mRNAs encoding two other secreted proteins, fibronectin (FIB) and matrix metalloproteinase 12 (MMP12) (Fig 1A). Actin mRNA was equally distributed between the cytosol and membrane. Similar dual distribution of several mRNAs encoding cytosolic proteins has been observed before [52,40] and we used actin mRNA as additional control in our experiments. Real time RT-PCR measurement of the mRNA distribution is shown later. When the cells were treated with pateamine A (Fig 1B) or puromycin (Fig 1C), the membrane association of collagen α1(I) and α2(I) mRNAs remained unchanged, however, the localization of FIB and MMP12 mRNAs was dramatically altered. About 40% of MMP12 mRNA (for real time RT-PCR measurement see later) and 10–20% of fibronectin mRNA were released into the cytosol. Distribution of actin mRNA was unchanged.

Analysis of ribosomal RNA in the fractions revealed that in HLF about 80% of ribosomes are associated with the membrane (Fig 1A). This was expected, as HLF are specialized cells dedicated to secretion of extracellular matrix proteins. Similar, predominantly membrane distribution of ribosomal RNA, was found in other cell types that secrete large amounts of proteins, including plasmocytoma cells and mouse embryonic fibroblasts [53]. Most ribosomes were released from the membrane by treatment with pateamine A or puromycin (Fig 1B and C), as evidenced by the shift of ribosomal RNA into the cytosolic fraction. To verify that there was no cross-contamination of the fractions and no release of the nuclear material we analyzed the following proteins; calnexin as ER marker, GAPDH as cytosolyc marker and histone H4 nuclear marker (Fig 1A, B, C and D, bottom panels). The analysis demonstrated that the fractions were devoid of significant contamination. These results suggested that collagen mRNAs remain associated with the internal membranes in the absence of translation and that they may attach to the ER membrane differently than the mRNAs encoding other secreted proteins. Treatment with cycloheximide did not release FIB, MMP12 or collagen mRNAs from the membranes (Fig 1D). The analysis of ribosomal RNA revealed that most ribosomes remained attached to the membrane, suggesting that, when polysomes were immobilized, the membrane association of FIB and MMP12 mRNAs was retained.

A time course experiment (Fig 1E) revealed that collagen mRNAs remain associated with the membranes 12 h after the translation inhibition by Pat-A, with only tracing amounts of collagen α2(I) mRNA released after 12 h. FIB and MMP12 mRNA were partially released after 3 h and progressively increasing amounts were released at subsequent time points (Fig 1E). This suggested that mRNAs encoding secreted proteins differ in the way they partition to the ER membrane and that collagen mRNAs stably associate with the internal membranes in the absence of translation.

### Association of collagen mRNAs with the ER membrane is mediated by LARP6

**Figure 2:**
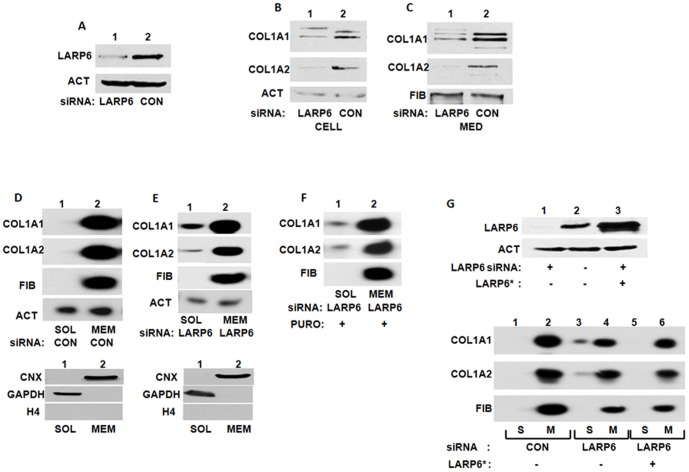
Knock down of LARP6 decreases expression of type I collagen. A. Knock down of LARP6. Expression of LARP6 was analyzed by western blot in HLFs after transfection of LARP6 siRNA (lane 1) and control siRNA (lane 2). Loading control: actin (ACT). B. Cellular level of collagen polypeptides after LARP6 knock down. Collagen α1(I) (COL1A1) and α2(I) (COL1A2) polypeptides were analyzed by western blot in LARP6 knock down HLFs (lane 1) and control HLFs (lane 2). C. Secretion of collagen polypeptides into the cellular medium. Cellular medium of cells in B was analyzed by western blot. Loading control: fibronectin (FIB). D. Partitioning of collagen mRNAs in control cells. Upper panel: HLFs transfected with control siRNA were fractionated into cytosolic (SOL) or membrane (MEM) fraction and analyzed for presence of collagen mRNAs and actin (ACT) and fibronectin (FIB) mRNAs by RT-PCR. Bottom panel: distribution of the marker proteins. E. Partitioning of collagen mRNAs in LARP6 knock down cells. Experiment as in D, except LARP6 siRNA was transfected. F. Partitioning of collagen mRNAs in LARP6 knock down cells after puromycin treatment. Experiment as in E, except the cells were treated with puromycin for 3 h. G. Rescue of partitioning of collagen mRNAs by supplementing LARP6. Upper panel: expression of LARP6 in LARP6 knock down cells (lane 1), in control cells (lane 2) and in LARP6 knocked down cells transduced with LARP6 adenovirus (lane 3). Loading control: actin (ACT). Bottom panel: Distribution of collagen mRNAs in the soluble (S) and membrane (M) fractions of control cells (lanes 1 and 2), LARP6 knock down cells (lanes 3 and 4) and LARP6 knock down cells supplemented with exogenous LARP6 (lanes 5 and 6).

To investigate if LARP6, as collagen mRNA specific RNA binding protein [14], is required for translation independent association of collagen mRNAs with the ER membrane, we knocked down LARP6 and analyzed the partitioning of collagen mRNAs. Using siRNA we were able to knock down LARP6 in lung fibroblasts by ∼80% (Fig 2A). When LARP6 was knocked down, the expression of collagen α1(I) and α2(I) polypeptides was reduced when the peptides was measured either intracelullarly (Fig 2B) or secreted into the medium (Fig 2C). Thus, the cells with reduced amounts of LARP6 inefficiently synthesize type I collagen.

When partitioning of collagen mRNAs between soluble and membrane fractions was analyzed in control cells, collagen α1(I) and α2(I) mRNAs were found almost entirely in the membrane fraction, as before (Fig 2D). The purity of the fractions is shown at the bottom of Figs 2D and 2E. However, in LARP6 knock down cells, a significant amount of collagen α1(I) mRNA and lesser amounts of α2(I) mRNA were found free in the cytosol (Fig 2E). The partitioning of fibronectin mRNA and actin mRNA was not affected. For real time RT-PCR measurement of the mRNA distribution, including the MMP12 mRNA, see later. Thus, the reduced amounts of LARP6 altered the subcellular partitioning of collagen α1(I) mRNAs, with a marginal effect on collagen α2(I) mRNA. This suggested that the decreased production of collagen polypeptides in LARP6 knock down cells (Fig 2B and C) may be related to the impaired association of collagen mRNAs with the ER membrane.

To further corroborate that translation is dispensable for membrane association of collagen mRNAs we inhibited translation by puromycin in LARP6 knock down cells (Fig 2F). No additive effect was observed and partitioning of collagen mRNAs was similar to that in LARP6 knock down cells in the presence of translation (compare Figs 2E and 2F).

Partitioning of collagen mRNAs was rescued when LARP6 was supplemented to the LARP6 knock down cells. Fig 2G, upper panel, shows knock down of LARP6 in HLF (lane 1) and the level of the protein in control cells (lane 2). Delivery of LARP6 by an adenovirus into the LARP6 knock down cells (LARP6*, lane 3) resulted in restoration of the LARP6 levels. This completely rescued the partitioning of collagen mRNAs (Fig 2G, bottom panel), which were now found exclusively in the membrane fraction (compare lanes 3 and 4 to lanes 5 and 6).

### Altered modifications of collagen polypeptides in LARP6 knock down cells

**Figure 3:**
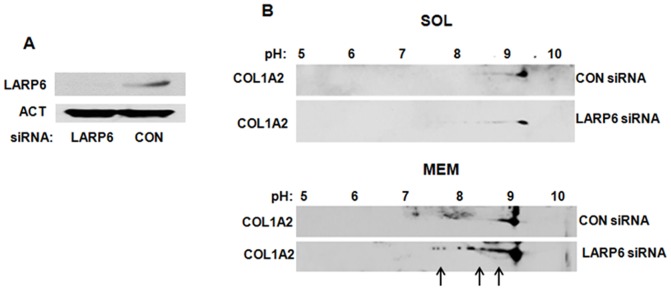
Collagen modifications in LARP6 knock down cells. A. Knock down of LARP6. Expression of LARP6 in HLFs transfected with LARP6 siRNA (lane 1) and control siRNA (lane 2). Loading control: actin (ACT). B. Modifications of collagen α2(I) polypeptides. HLFs transfected with control siRNA (CON) or LARP6 siRNA were fractionated into cytosolic (SOL, upper panels) and membrane (MEM, lower panels), the fractions were resolved by 2D SDS-PAGE gels and collagen α2(I) polypeptide (COL1A2) visualized by western blotting. Numbers indicate the pH range of the isoelectric focusing strips and excessive collagen modifications are indicated by arrows.

Prior to folding into the triple helix collagen polypeptides must be modified by hydroxylations of selected prolines and lysines and glycosylation of hydroxy-lysines [1], [2]. Hypermodifed collagen polypeptides appear when the rate of translation is not coupled to the rate of folding [54], [55], [56], [57]. Therefore, we analyzed the modifications of collagen polypeptides present in soluble and membrane fractions after knocking down LARP6 using 2D SDS-PAGE and western blotting. The available antibody recognized only α2(I) polypeptide in 2D SDS-PAGE gels, so we could only show the results for this polypeptide. In cytosolic fraction of control cells collagen α2(I) polypeptide was found in small amounts and was presented as relatively homogeneous molecules, isoelectrically focused around pH 9.2. The knock down of LARP6 did not change their abundance or pI (lower panel) (Fig 3B, upper panels). In the membrane fraction of control cells α2(I) polypeptide was resolved as molecular species having isoelectric point from 8.8–9.1 with majority focusing at pH 9.1. In LARP6 knock down cells, an increased fraction of molecules focused between pH 7.5 and 8.8 (Fig 3B, lower panels, arrows), suggesting additional modifications that changed the pI. Although, we could not discern the nature of modifications causing the pI shift, it is known that hydroxylations of amino-acids shift the pI to a more acidic region [58], [59]. Therefore, we assumed that the additional modifications represent excessive hydroxylations of prolines and lysines. This indicated that knock down of LARP6, not only decreases the total collagen synthesis, but also results in hypermodifications of individual polypeptides. Thus, direct targeting of collagen α1(I) mRNA to the ER membrane, and to a lesser extent of α2(I) mRNA, by LARP6 is critical for efficient synthesis of type I collagen, including proper posttranslational modifications.

### Integrity of nonmuscle myosin filaments as requirement for collagen synthesis

**Figure 4:**
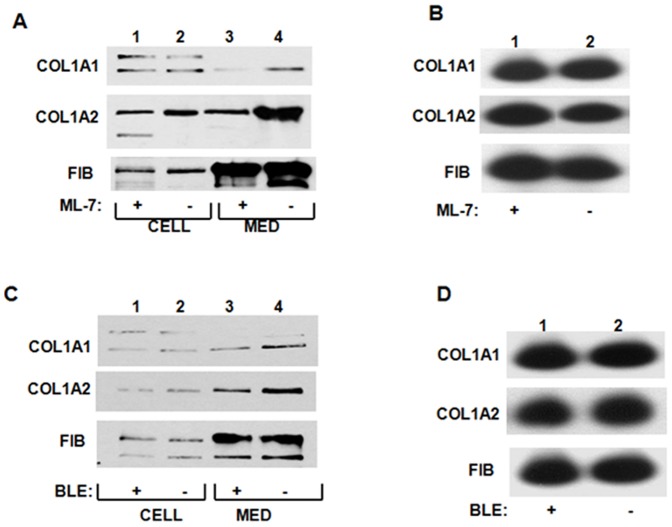
Disruption of nonmuscle myosin filaments decreases type I collagen production. A. Effect of ML-7. HLFs were treated with ML-7 and the level of collagen polypeptides intracellularly (lanes 1 and 2) or secreted into the culture medium (lanes 3 and 4) was analyzed by western blot. Loading control: fibronectin (FIB). B. ML-7 does not affect expression of collagen mRNAs. Total RNA was extracted from cells in A and analyzed for expression of collagen and fibronectin mRNAs by RT-PCR. C. Effect of blebbistatin. Experiment as in A, except HLFs were treated with blebbistatin. D. Expression of collagen mRNAs in cells treated with blebbistatin. Experiment as in C, except the cells were treated with blebbistatin.

Our previous work suggested that the integrity of nonmuscle myosin filaments is necessary for synthesis of type I collagen [15], [33], while experiments in mice showed that cardiac fibrosis is found only in the regions of the heart re-expressing nonmuscle myosin [60]. Here we wanted to investigate if nonmuscle myosin filaments participate in the membrane targeting of collagen mRNAs. To this end we depolymerized the filaments using ML-7, a specific inhibitor of myosin light chain kinase (MLCK) [61], [62]. Additionally, we inactivated the motor function of myosin by blebbistatin, an inhibitor of the myosin ATPase activity [63], to compare the requirement for the integrity of the filaments versus their motor function. Neither of the inhibitors changed the level of collagen mRNAs (Fig 4B and D), suggesting that transcription or stability of collagen mRNAs was not affected. Treatment with ML-7 decreased the amount of α1(I) and α2(I) polypeptides secreted into the cellular medium, while the effect on the cellular level was minimal (Fig 4A), suggesting that a defect may be in the folding of collagen triple helix or its secretion into the medium. Treatment with blebbistatin had a weaker effect; the amount of collagen α1(I) and α2(I) polypeptides secreted into the cellular medium was affected less by this drug than by ML-7 (Fig 4C).

**Figure 5:**
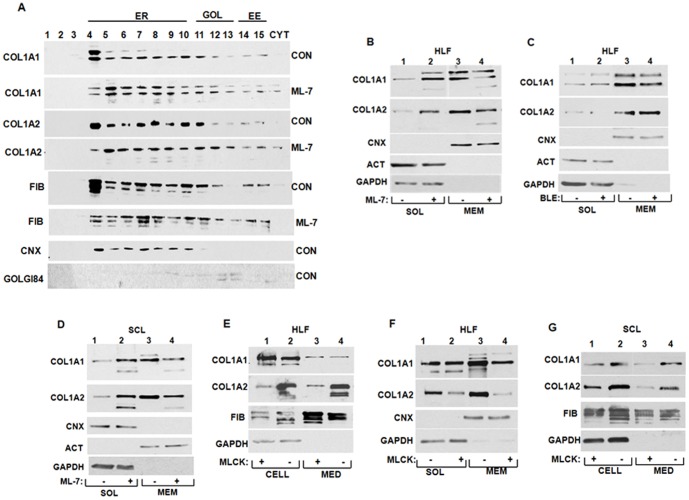
Disruption nonmuscle myosin results in cytosolic accumulation of collagen polypeptides. A. Distribution of collagen polypeptides in the secretory pathway compartments. Extracts of control and ML-7 treated HLFs were fractionated by density gradient centrifugation into fractions containing endoplasmic reticulum (ER), Golgi (GOL), early endosomes (EE) or cytosol (CYT) and the fractions were analyzed by western blot for presence of collagen α1(I) (COL1A1), collagen α2(I) (COL1A2) and fibronectin (FIB) polypeptides. Calnexin (CNX) was analyzed as a marker of the ER and golgin 84 (GOLGI84) as a marker of the Golgi complex. B. Cytosolic accumulation of collagen polypeptides after disruption of nonmuscle myosin in HLF. HLF were treated with ML-7 and fractionated into cytosolic (SOL, lanes 1 and 2) and membrane (MEM, lanes 3 and 4) fractions. The fractions were analyzed for collagen α1(I) (COL1A1), collagen α2(I) (COL1A2) polypeptides by western blot. Calnexin (CNX) was analyzed as a marker of membrane fraction and actin (ACT) and GAPDH as a markers of cytosolic fraction. C. Inhibition of the motor function of nonmuscle myosin has little effect on cytosolic accumulation of collagen polypeptides. Experiment as in B, except HLFs were treated with blebbistatin. D. Cytosolic accumulation of collagen polypeptides after disruption of nonmuscle myosin in scleroderma fibroblasts. Experiment as in B, except human scleroderma fibroblasts were used. E. Inhibition of collagen expression by DN-MLCK. DN-MLCK was overexpressed in HLFs and collagen α1(I) (COL1A1) and α2(I) (COL1A2) polypeptides were analyzed in cell extract (lanes 1 and 2) and in cellular medium (lanes 3 and 4). Loading controls; fibronectin (FIB) and GAPDH. F. Cytosolic accumulation of collagen polypeptides after disruption of nonmuscle myosin by DN-MLCK. DN-MLCK was overexpressed in HLF and cells were fractionated into cytosolic (SOL, lanes 1 and 2) and membrane (MEM, lanes 3 and 4) fractions. The fractions were analyzed for collagen α1(I) (COL1A1) and collagen α2(I) (COL1A2) polypeptides by western blot. Loading controls; calnexin (CNX) and GAPDH. G. Inhibition of collagen expression by DN-MLCK in scleroderma fibroblasts. DN-MLCK was overexpressed in scleroderma fibroblasts and collagen α1(I) (COL1A1) and α2(I) (COL1A2) polypeptides were analyzed in cell extract (lanes 1 and 2) and in cellular medium (lanes 3 and 4). Loading controls; fibronectin (FIB) and GAPDH.

To distinguish if ML-7 treatment affects folding of collagen triple helix or its secretion into the medium we first analyzed distribution of collagen polypeptides throughout the secretory pathway. We separated ER from Golgi and early endosomes by centrifugation through iodixanol gradient [41] and analyzed the presence of collagen polypeptides in the fractions by western blot. We exposed the western blots to achieve similar signal intensity of control and ML-7 treated cells to directly compare the relative distribution of collagen polypeptides in the fractions. By analyzing calnexin (ER marker) [64] and Golgin84 (Golgi marker) [65], we assigned the fractions representing these compartments and assigned fractions containing early endosomes according to [41]. As shown in Fig 5A, the relative distribution of collagen α1(I) and α2(I) polypeptides throughout the secretory pathway was similar in cells treated with ML-7 and in control cells. The major difference was fraction 4, where control cells had significantly more collagen than ML-7 treated cells. However, fraction 4 also contained more fibronectin in ML-7 treated cells, which was normally secreted. Therefore, we concluded that the alteration of fraction 4 is not a likely cause of poor secretion of type I collagen. The relatively unperturbed distribution of collagen polypeptides throughout the secretory pathway suggested that collagen polypeptides that have entered into the pathway are transported normally between the compartments. However, we noticed that both collagen polypeptides were present in increased amount in the cytosol (Fig 5A, CYT fraction) in the ML-7 treated cells. This suggested that some collagen polypeptides accumulate in the cytosol when nonmuscle myosin filaments were disrupted.

To further investigate this phenomenon we fractionated cells in soluble and membrane fractions and analyzed the level of collagen polypeptides by western blot. In ML-7 treated HLFs, the level of both collagen polypeptides decreased by ∼50% in the membrane fraction and increased by ∼50% in the soluble fraction, compared to control cells (Fig 5B). ML-7 treatment did not change the distribution of calnexin, actin or GAPDH proteins. This verified that depolimerization of nonmuscle myosin filaments results in specific redistribution of type I collagen from the membrane compartment into the cytosol. Blebbistatin treatment did not increase the cytosolic retention of collagen polypeptides (Fig 5C), what is consistent with a minimal effect on their secretion (Fig 4C). Thus, integrity of the nonmuscle myosin filaments, rather than their motor function, appears to be critical for proper secretion of type I collagen.

To verify that a similar requirement holds for other collagen producing cells we analyzed human scleroderma skin fibroblasts (Fig 5D). About 50% of collagen polypeptides was shifted into the soluble fraction when scleroderma fibroblasts were treated with ML-7, suggesting that myosin dependent collagen production is a general characteristic of collagen producing cells.

To exclude the nonspecific effects of ML-7 we disrupted nonmuscle myosin by overexpressing dominant negative isoform of myosin light chain kinase (DN-MLCK) [33]. Myosin light chain kinase (MLCK) phosphorylates regulatory light chains of nonmuscle myosin to promote assembly of the filaments [66]. A dominant negative isoform of MLCK (DN-MLCK) was developed [34], which inhibits MLCK and which has been used in previous studies to disrupt nonmuscle myosin filaments [34], [67], [68]. Overexpression of DN-MLCK in HLFs decreased the secretion and intarcellular level of collagen α2(I) polypeptide, while the secretion of collagen α1(I) polypeptide was unaltered (Fig 5E, lanes 1 and 3). A strong effect of DN-MLCK on α2(I) polypeptide and a minimal effect on α1(I) polypeptide was reported before for HLFs and was attributed to the ability of HLFs to excrete homotrimers of α1(I) polypeptides in myosin independent manner [33]. When the intracellular distribution of collagen polypeptides between soluble and membrane fraction was analyzed (Fig 5F), DN-MLCK caused predominantly cytosolic accumulation of α2(I) polypeptide and slightly increased cytosolic retention of α1(I) polypeptide (compare lanes 2 and 4). Overexpression of DN-MLCK in scleroderma fibroblasts resulted in decrease in expression of both collagen polypeptides (Fig 5G). These results corroborated the findings using ML-7 and supported the notion that integrity of nonmuscle myosin filaments is a prerequisite for effective type I collagen synthesis.

### Collagen polypeptides retained in the cytosol in absence of nonmuscle myosin filaments are hypermodified

**Figure 6:**
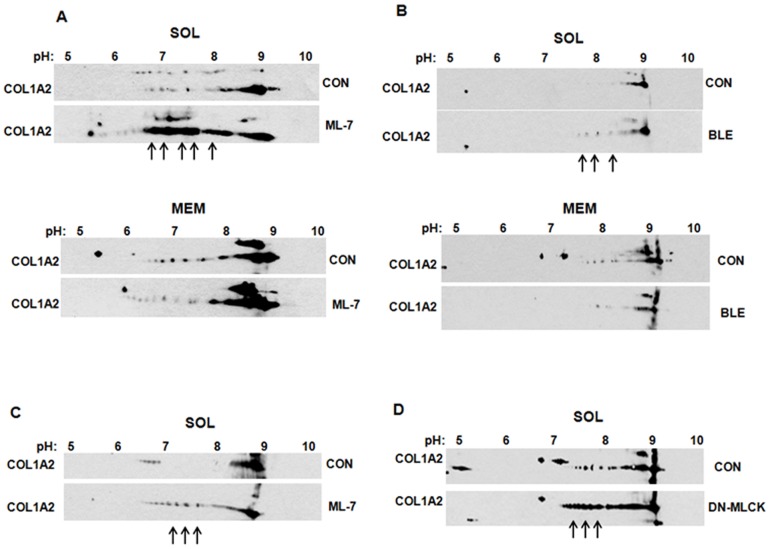
Hypermodifications of collagen α2(I) polypeptides accumulated in the cytosol after disruption of nonmuscle myosin. A. Modifications of collagen α2(I) polypeptides accumulated in the cytosol after ML-7 treatment. Control HLFs (CON) and HLF treated with ML-7 were fractionated into cytosolic (SOL) and membrane (MEM) fractions and the fractions were analyzed by 2D SDS-PAGE, followed by western blotting. The pH range of isoelectric focusing strips is shown on the top and excessive modifications of collagen α2(I) polypeptide are indicated by arrows. B. Same experiment as in A, except the cells were treated with blebbistatin. C. Experiment as in A, except scleroderma skin fibroblasts were used and only cytosolic (SOL) fraction is shown. D. DN-MLCK was overexpressed in HLFs and collagen α2(I) polypeptide was analyzed in the cytosolic fraction (SOL) of control and DN-MLCK expressing cells by 2D SDS-PAGE and western blotting. Arrows indicate the hypermodifications.

Unfolded or grossly aberrant collagen polypeptides are retro-translocated from the ER into the cytosol for degradation [57], [69], [70]. Appearance of increased amount of collagen polypeptides in the cytosol upon inactivation of nonmuscle myosin suggested that these polypeptides may have been retro-translocated from the secretory pathway and that their abundance may have saturated the degradation machinery. Therefore, we analyzed the posttranslational modifications of collagen α2(I) polypeptide found in the cytosol of ML-7 treated cells by 2D SDS-PAGE and western blotting (Fig 6A). Upper panels show cytosolic fractions and lower panels show membrane fractions of control cells and of cells treated with ML-7. For better qualitative comparison, the western blot of cytosolic fraction of control cells was exposed much longer to achieve a similar signal to that of ML-7 treated cells. α2(I) polypeptide found in the cytosol of ML-7 treated cells appeared massively hyper-modified, as >50% of molecules were shifted into the more acidic region between pH of 6.5 and 8 (arrows in Fig 6A). α2(I) polypeptide in control cells had the pI around 9, as observed before (Fig 3). As hydroxylations typically change the pI towards more acidic region [58], and an acidic shift of hyper-hydroxylated collagen polypeptides has been reported in a patient with osteogenesis imperfecta [59], we again assumed that excessive hydroxylations of prolines and lysines represented the over-modifications. The isoelectric focusing of α2(I) polypeptide in the membrane fraction of ML-7 treated cells showed similar pattern to that in control cells (Fig 6A, lower panels). The most molecules in both cell types had a pI from 8 to 9, suggesting that almost all of hypermodified collagen polypeptides in the ML7 treated cells were translocated into the cytosol. We concluded that grossly abnormal collagen polypeptides are synthesized when nonmuscle myosin filaments are disrupted and that the hypermodified polypeptides are eliminated from the secretory pathway. This is in contrast with LARP6 knock down cells, where hypermodified collagen polypeptides were primarily found in the membrane fraction. The analysis of scleroderma fibroblasts verified that ML-7 has a similar effect in cells of different origin (Fig 6C).

Treatment with blebbistatin also resulted in cytosolic accumulation of hypermodified α2(I) polypeptides, but the extent of modifications and the fraction of molecules affected was much smaller (Fig 6B), again suggesting that the motor function of nonmuscle myosin is not as critical for collagen synthesis as the integrity of the filaments.

When nonmuscle myosin filaments were disrupted using DN-MLCK [34], hypermodified collagen α2(I) polypeptides also accumulated in the cytosol (Fig 6D), resembling the effects of ML-7. Thus, this result further confirmed that hypermodifications of collagen polypeptides are specifically due to disruption of nonmuscle myosin filaments and not to unanticipated effects of ML-7.

Although we could not analyze collagen α1(I) polypeptide due to lack of its recognition in 2D gels by the antibody available, we concluded that inactivation of nonmuscle myosin results in massive hyper-modifications of collagen polypeptides, retention of the hyper-modified molecules in the cytosol and diminished secretion of type I collagen and that these events were not associated with a general disruption of the secretory pathway.

### Role of nonmuscle myosin in membrane partitioning of collagen mRNAs

**Figure 7:**
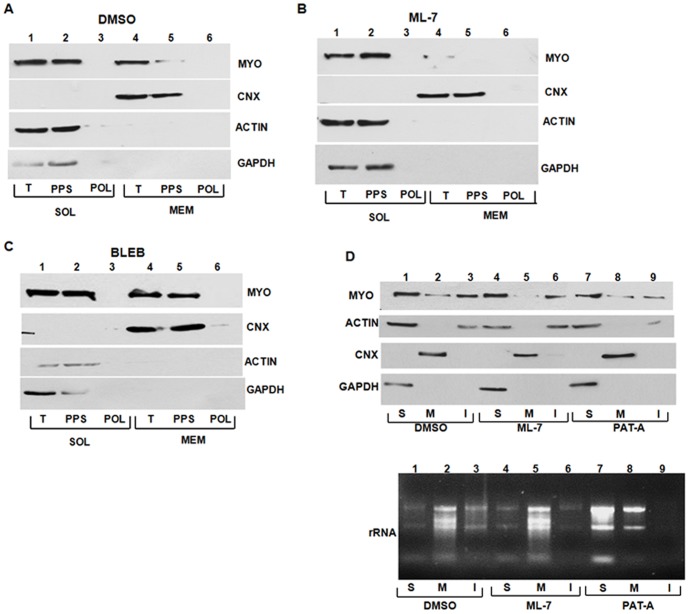
Association of nonmuscle myosin with internal membranes. A. Distribution of nonmuscle myosin in cytosolic and membrane fractions of HLFs. HLFs were fractionated into cytosolic (SOL) and membrane (MEM) fractions and the total fractions (T) were analyzed in lanes 1 and 4. Polysomes (POL, lanes 3 and 6) and post-polysomal supernatant (PPS, lanes 2 and 5) were furthered separated from the total fractions and the samples were analyzed for myosin IIb (MYO) by western blot. Calnexin (CNX) was analyzed as a marker of membrane fraction and actin (ACT) and GAPDH as a markers of cytosolic fraction. B. Disappearance of nonmuscle myosin from the membrane fraction after ML-7 treatment. Experiment as in A, except HLFs were treated with ML-7. C. Effect of blebbistatin on membrane association of myosin IIb. Experiment as in A, except HLFs were treated with blebbistatin. D. ML-7 treatment does not increase insoluble nonmuscle myosin. Upper panel: distribution of nonmuscle myosin (MYO), actin (ACT), calnexin (CNX) and GAPDH proteins in soluble (S), membrane (M) and NP-40 insoluble (I) fractions of control cells (lanes 1–3), ML-7 treated cells (lanes 4–6) and Pat-A treated cells (lanes 7–9). Bottom panel: distribution of ribosomal RNA.

We reasoned that hyper-modifications of α2(I) polypeptide caused by ML-7 treatment may also be due to perturbed partitioning of collagen mRNAs to the ER membrane. To test this we first analyzed if nonmuscle myosin is associated with the internal membranes. Using sequential detergent extraction we found that ∼60% of nonmuscle myosin was extractable in the membrane fraction, while ∼40% was found in the cytosol (Fig 7A, lanes 1 and 4). To assess if the membrane bound nonmuscle myosin supports polysomes formation, we further separated cytosolic and membrane fractions into polysomes and postpolysomal supernatant. In the cytosolic fraction nonmuscle myosin did not copurify with polysomes; all of it was found in the post polysomal supernatant (Fig 7A, lanes 2 and 3). A similar distribution was found in the membrane fraction, where all nonmuscle myosin was present in the post polysomal supernatant (Fig 7A, lanes 5 and 6). When cells were treated with ML-7, most of the nonmuscle myosin in the membrane fraction disappeared (Fig 7B, compare lanes 1 and 4), suggesting that depolymerization of the filaments causes detachment of the myosin from the membranes. Treatment with blebistatin did not disrupt the association of nonmuscle myosin with the membranes (Fig 7C, lanes 4 and 5). About 50% of nonmuscle myosin was found associated with membranes in control and blebbistatin treated cells.

To exclude the possibility that depolymerization of nonmuscle myosin by ML-7 may have caused its sequestration into insoluble material, rather than its release from the membrane, we fractionated the cells as before. However, we also extracted the cell remnants with 1% SDS to solubilize the proteins that were insoluble in NP-40 used for the membrane extraction and analyzed all the fractions by western blot. Fig 7D shows that in untreated cells a significant fraction of nonmuscle myosin was found in the NP-40 insoluble fraction (lane 3). Importantly, ML-7 treatment did not increase the amount of insoluble myosin (lane 6), but decreased the amount associated with the membrane (lane 5). Pat-A treatment did not significantly alter the fractionation of nonmuscle myosin (lanes 7–9).

To assess if ML-7 causes retention of ribosomes in the insoluble fraction, we extracted total RNA from all fractions and analyzed it by agarose gel electrophoresis (Fig 7D, bottom panel). In control cells most ribosomal RNA was found in the membrane fraction (lane 2), but a significant portion was also found in the NP-40 insoluble fraction (lane 3). ML-7 did not change such distribution of ribosomal RNA. In contrast, in Pat-A treated cells most ribosomal RNA was found in the cytosol and it disappeared from the NP40 insoluble fraction. This suggested that ML-7 treatment did not cause significant sequestration of nonmuscle myosin and ribosomes into the insoluble material, but that inhibition of translation initiation releases polysomes from the membranes and from the NP-40 insoluble proteins.

**Figure 8:**
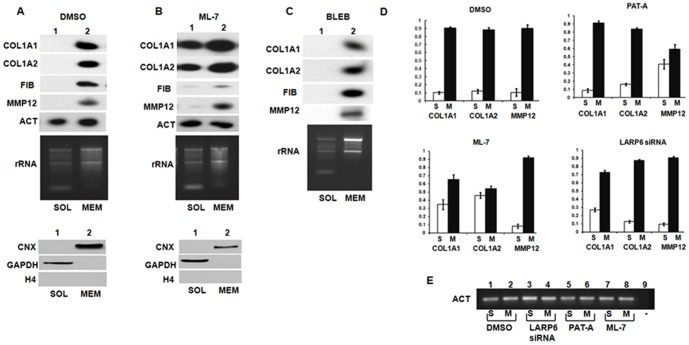
Membrane partitioning of collagen mRNAs after disruption of nonmuscle myosin. A. Partitioning of collagen mRNAs in control cells. Upper panel: HLFs were fractionated in cytosolic fraction (SOL, lane 1) or membrane fraction (MEM, lane 2) and collagen α1(I) (COL1A1), collagen α2(I) (COL1A2), fibronectin (FIB), matrix metalloproteinase 12 (MMP12) and actin (ACT) mRNAs were analyzed by RT-PCR. Distribution of ribosomal RNA (rRNA) is shown by agarose gel electrophoresis and ethidium bromide staining. Bottom panel: distribution of calnexin (CNX), GAPDH and histone H4 proteins in the fractions, analyzed by western blot. B. Partitioning of collagen mRNAs in ML-7 treated cells. Experiment as in A, except HLFs were treated with ML-7. C. Partitioning of collagen mRNAs in blebbistatin treated cells. Experiment as in A, except HLFs were treated with blebbistatin. D. Real time RT-PCR determination of collagen mRNAs. The treatment of HLFs is indicated above the panels. The cells were fractionated into cytosolic (S) and membrane (M) fractions, RNA was extracted and analyzed by real time RT-PCR for presence of collagen α1(I) (COL1A1), collagen α2(I) (COL1A2) and matrix metalloproteinase 12 (MMP12) mRNAs. The signals were normalized to actin mRNA, which was found in similar amounts in S and M fractions. The normalized expression of each mRNA in S and M fractions was summed up and arbitrarily set as 1. Bars represent the relative proportion of mRNAs in the fractions. Error bars: +- 1 SEM. E. Distribution of internal control actin mRNA. cDNA used in real time RT-PCR reactions in D was analyzed by PCR with primers specific for actin mRNA (ACT). Lanes 1 and 2, control cells, lanes 3 and 4, cells transfected with LARP6 siRNA, lanes 5 and 6, cells treated with Pat-A, lanes 7 and 8, cells treated with ML-7. Lane 9, control reaction without cDNA.

Having established that nonmuscle myosin is bound to the internal membranes, but does not support polysomes, we assessed if it plays a role in partitioning of collagen mRNAs. When the cells were treated with ML-7 ∼40% of collagen α1(I) mRNA and α2(I) mRNA were released from the membrane and were found free in the cytosol (Fig 8B), compared to control cells in which all collagen mRNAs were found associated with the membrane (Fig 8A). Partitioning of FIB, MMP12 and actin mRNAs was minimally altered, suggesting that mRNAs encoding other secreted proteins do not require intact nonmuscle myosin for their association with the ER membrane. Figure 8D summarizes the quantitative RT-PCR determinations of collagen mRNAs distributed between soluble and membrane fractions of cells treated with Pat-A, ML-7 or transfected with LARP6 siRNA. Since actin mRNA was used for normalization of the real time RT-PCR results, we verified that distribution of actin mRNA was unaltered upon ML-7, Pat-A and LARP6 siRNA treatment (Fig 8E). Most of the ribosomal RNA also remained bound to the membrane after ML-7 treatment, indicating no major rearrangement of the translation machinery at the ER membrane, as predicted by the lack of association of nonmuscle myosin with polysomes. Blebbistatin did not have any effect of partitioning of collagen mRNAs (Fig 8C). This is consistent with a minimal effect of this drug on modifications of collagen polypeptides (Fig 6B), their secretion or cytosolic retention (Fig 5C).

From these experiments we concluded that the integrity of nonmuscle myosin filaments is required for direct membrane targeting of collagen mRNAs. In the absence of myosin dependent collagen mRNA partitioning the cells poorly secrete type I collagen and accumulate large amount of hyper-modified collagen polypeptides in the cytoplasm.

## Discussion

Understanding excessive synthesis of type I collagen in fibrosis is a key to find a cure for this intractable condition. Random translation of collagen mRNAs and their targeting to the ER membrane by the signal recognition particle (SRP) can not account for the most features of type I collagen synthesis. In reparative and reactive fibrosis collagen producing cells upregulate collagen biosynthesis several hundred fold [11], [12], [13], [71], [72], [73]. Heterotrimeric type I collagen is synthesized, without formation of the homotrimers of α1(I) polypeptides. Stable homotrimers of α1(I) polypeptides readily form when α2(I) polypeptide is not available [74], [75], [76], [77], but their complete absence suggests tightly regulated shunting of α2(I) polypeptide into the biosynthetic pathway. If folding of collagen polypeptides into the heterotrimer is delayed, the polypeptides are hyper-modified and subjected to intracellular degradation [54], [55], [56]. This suggests that there is a kinetic equilibrium between translation, posttranslational modifications and folding and excludes random processes. Folding of collagen heterotrimer is highly concentration dependent, as mutual recognition of three molecules initiates the event. When collagen folding was mimicked in vitro using model peptides, the triple helix was formed at mM concentrations of the peptides [78], [79]. It is not possible to achieve such high concentrations of collagen polypeptides within the lumen of the ER, unless the synthesis of α1(I) and α2(I) polypeptides is concentrated at discrete sub-regions. Molecular chaperones facilitate protein folding in vivo, but the only collagen specific chaperone is HSP48. HSP48 acts on all collagens and it has been demonstrated that HSP48 prevents lateral aggregation of the collagen trimers and does not facilitate their formation [70], [80], [81].

We postulate here the existence of a mechanism that facilitates type I collagen synthesis by directly targeting collagen mRNAs to the membrane of the ER. We show that: 1. mRNAs encoding type I collagen are associated with the ER membrane in the absence of translation. 2. LARP6, which specifically binds 5′SL of collagen mRNAs, is involved in this process. 3. Intact nonmuscle myosin filaments, rather than their motor function, are needed for association of collagen mRNAs with the ER membrane. 4. When partitioning of collagen mRNAs to the ER membrane is disrupted, collagen polypeptides become hyper-modified and are poorly secreted. Thus, to efficiently synthesize, modify and fold type I collagen it is critical to directly partition collagen mRNAs to the ER membrane.

Partitioning of mRNA to the ER membrane is believed to be mediated by RNA binding proteins [44], [46], [48]. Rrbp1 (ribosome binding protein 1, p180) is ribosomal receptor on the ER membrane [82]. Depletion of Rrbp1 results in decreased association of ribosomes with the ER membrane and poor secretion of type I collagen [83]. This suggests that Rrbp1 facilitates translation at the ER membrane. It has been reported that Rrbp1 promotes translation independent localization of subset of mRNAs to the ER membrane by binding the RNA through its lysine rich domain, although Rrbp1 does not bind RNA in a sequence specific manner [48]. Ribosome independent maintanence of placental alkaline phosphatase mRNA and calreticulin mRNAs was mediated by Rrbp1 [84]. It is possible that Rrbp1 also participates in partitioning of collagen mRNAs, together with LARP6 and nonmuscle myosin, and this remains to be explored. Knock down of LARP6 resulted in dissociation of collagen α1(I) mRNA from the membrane, but the effect on α2(I) mRNA was negligible (Figs 2 and 8D). However, in our experiments the knock down was not complete and our preliminary results suggests that binding of LARP6 to the 5′SL of collagen α2(I) mRNA is more stable than binding to the α1(I) 5′ SL. Thus, the residual LARP6 may have been sufficient to support membrane partitioning of α2(I) mRNA, while that of α1(I) mRNA was partially compromised. Making mutations in the 5′SL of collagen α1(I) and α2(I) mRNAs or creating total LARP6 knock cells is needed to fully understand the LARP6 independent partitioning. Nevertheless, this suggested that translation independent partitioning of collagen mRNAs to the ER membrane is partially mediated by LARP6. LARP6 binds 5′ SL of collagen mRNAs with high affinity and the binding overlaps with the start codon. We hypothesize that LARP6 binding delays initiation of translation until collagen mRNAs are targeted to the ER. Overexpression of LARP6 inhibits translation of collagen mRNAs [14], indicating that LARP6 can act as a translational inhibitor. It has been reported that synthesis of type I collagen is not uniform throughout the ER, but concentrated at discrete loci [14], [33]. Direct targeting to such specialized loci would increase the local concentration of α1(I) and α2(I) mRNAs, coordinate the synthesis of nascent collagen polypeptides, couple posttranslational modifications with folding and facilitate incorporation of the α2(I) polypeptide into the heterotrimer. The proteins which define the loci of collagen biosynthesis on the ER membrane are not completely characterized. Translocation associated membrane protein 2 (TRAM2) has been found associated with translocons engaged in synthesis of collagen polypeptides [85]. TRAM2 recruits the Ca++ pump of the ER, Serca2b, to these translocons and it has been postulated that the increased Ca++ concentrations stimulate molecular chaperones to fold collagen heterotrimers [85]. If there are other proteins besides TRAM2 that define the putative collagen specific translocons, remains to be discovered.

In cardiac fibrosis, the areas of fibrosis were found only in the regions of the heart re-expressing nonmuscle myosin [60] and we have reported nonmuscle myosin dependent collagen synthesis in fibroblasts [33]. The results shown here indicate that intact filaments composed of nonmuscle myosin are necessary for the process of direct partitioning of collagen mRNAs. A large fraction of nonmuscle myosin can be extracted from the intracellular membranes (Fig 7). It has been reported that nonmuscle myosin associates with Golgi membranes [86] and that it also propels terminal vesicles loaded with pre-assembled collagen fibrils out of the cell [4]. Here we show that the secretory pathway through ER and Golgi is not affected by depolimerization of nonmuscle myosin (Fig 5). Instead, partitioning of collagen mRNAs to the ER membrane is perturbed, suggesting the role of nonmuscle myosin in this earliest step of the biosynthetic pathway (Fig 8). We have previously reported that LARP6 interacts with nonmuscle myosin [33], so it is possible that nonmuscle myosin filaments provide structural support for the components of the mechanism involved in partitioning of collagen mRNAs. Blebbistatin, an inhibitor of the motor function of myosin, has much smaller effect then ML-7, suggesting that the filament integrity, rather than their locomotor activity is essential.

The effect of disruption of nonmuscle myosin on collagen synthesis appears to be greater than knock down of LARP6. ML-7 treatment caused massive accumulation of hypermodified collagen polypeptides in the cytosol (Fig 6), a clear indication of dramatically perturbed collagen synthesis [56]. A similar result was obtained when nonmuscle myosin was disrupted by overexpression of DN-MLCK, excluding the nonspecific effects of ML-7 (Fig 5). When LARP6 was knocked down accumulation of hypermodified collagen was observed in the membrane compartment (Fig 3). The reason for this discrepancy is not clear and, although the residual LARP6 activity in the LARP6 knock down cells may have blunted the effect, it is likely that nonmuscle myosin may have additional roles in collagen biosynthesis.

Nonmuscle myosin has been profoundly studied in relation to cell motility and contractility [21], [26], [87], [88]. In fibrosis, quiescent fibroblasts or other collagen producing cells become activated by profibrotic cytokines, such as TGFβ [89], [90]. As part of the activation process the cells upregulate nonmuscle myosin expression and polymerize the filaments [30]; this allows their migration to the site of profibrotic injury. Our work implicates that formation of nonmuscle myosin filaments also allows the cells to partition collagen mRNAs to the ER membrane, as a prerequisite for productive synthesis of type I collagen. Thus, the ability of cells to migrate and the ability to produce type I collagen are two inseparable processes. Further characterization of their coupling will help understand the physiology of wound healing and pathogenesis of fibrosis.
